# *Medication reviews are useful, but the model needs to be changed*: Perspectives of Aboriginal Health Service health professionals on Home Medicines Reviews

**DOI:** 10.1186/s12913-015-1029-3

**Published:** 2015-09-10

**Authors:** Lindy Swain, Lesley Barclay

**Affiliations:** University Centre for Rural Health, University of Sydney, 55-61 Uralba Street, Lismore, NSW 2480 Australia

## Abstract

**Background:**

The Australian Home Medicines Review (HMR) program consists of a pharmacist reviewing a patient’s medicines at his or her home and reporting findings to the patient’s general practitioner (GP) to assist optimisation of medicine management. Previous research has shown that the complex HMR program rules impede access to the HMR program by Aboriginal and Torres Strait Islander clients.

This study explores the attitudes and perceptions of health professional employees working within Aboriginal Health Services (AHSs) towards the HMR program. The goal was to identify how the HMR program might better address the needs of Aboriginal and Torres Strait Islander people.

**Methods:**

Thirty-one semi-structured interviews were conducted with health professionals at 11 diverse AHSs. Fourteen Aboriginal Health Workers (AHWs), five nurses, one manager and 11 GPs were interviewed. Interviews were recorded, de-identified and transcribed verbatim. Transcripts were coded and analysed for themes that recurred throughout the interviews.

**Results:**

This study identified a number of barriers to provision of HMRs specific to Aboriginal and Torres Strait Islander clients. These included paternalistic attitudes of health professionals to clients, heightened protection of the GP-client relationship, lack of AHS-pharmacist relationships, need for more culturally responsive pharmacists and the lack of recognition of the AHS’s role in implementation of culturally effective HMRs.

Changes to the HMR model, which make it more effective and culturally appropriate for Aboriginal and Torres Strait Islander people, were recommended. Improved relationships between GPs and pharmacists, between pharmacists and AHSs, and between pharmacists and Aboriginal and Torres Strait Islander clients were identified as key to increasing HMRs for Aboriginal and Torres Strait Islander people.

**Conclusions:**

Aboriginal Health Services are well-placed to be the promoters, organisers, facilitators and implementers of health programs, such as HMR, for Aboriginal and Torres Strait Islander clients.

Embedding a pharmacist within an AHS addresses many of the barriers to HMRs. It ensures pharmacists are culturally mentored and that they build strong relationships with health professionals and clients.

The HMR program rules need to be changed significantly if medication review is to be an effective tool for improving medication safety and adherence for Aboriginal and Torres Strait Islander people.

## Background

The Australian Home Medicines Review (HMR) has been found to be an effective tool for improving medication safety, and reducing adverse events and unnecessary hospital admissions [1–3]. It consists of a pharmacist reviewing a patient’s medicines and reporting findings to the patient’s general practitioner (GP) to assist optimisation of medicine management. It is a ‘free to patient’, Australian Government managed program. An HMR referral is initiated by the patient’s GP and then an HMR-accredited pharmacist is organised to visit and interview the patient in his or her home. The pharmacist sends a report of findings to the GP, who then discusses recommendations and makes any appropriate medication changes in collaboration with the patient [4].

To claim funding from the Australian Government for an HMR the GP and pharmacist must adhere to program rules [4]. In the 2008 evaluation report [5] the complexity of business rules and the number of steps involved in the HMR process were identified as barriers to initiation of HMRs. The program rules stipulate the HMR referral can only be written by a GP. The GP must obtain the patient’s consent, a GP can only claim funding through the Medicare Benefits Scheme after a second visit from the patient to discuss the pharmacist’s HMR report and formulate the medication management plan, and the GP can only bill one out of the two consultations relating to the HMR. The suggested HMR referral form requires the GP to specify detailed patient information, and medical and medication history. The GPs often confuse the suggested indications on referral forms, such as taking five or more regular medications, with the specific rules for HMR program eligibility [5]. Rules state that a patient may only receive an HMR every 24 months or if a GP deems an HMR is specifically necessary due to significant changes to the patient’s condition or medication regimen. The latter part of this rule is rarely applied, for most GPs and pharmacists are concerned they will not receive payment if they step outside the specified 24 months. Thus, some eligible patients are not being referred for HMRs. The 24-month rule appears to have been applied due to budgetary restrictions of the program rather than as a result of any data that determine that this is an appropriate timeline for maximising medication management [6].

The HMR program rules and claim lodgement processes are also restrictive for pharmacists, as described below and as lamented by pharmacists in concurrent research [7]. The program’s rules have actually increased rather than decreased under the recent Fifth Government-Community pharmacy agreement [4]. The HMR payments can only by claimed by pharmacists if the HMR is conducted by an HMR-accredited pharmacist, if the patient is living in a community setting, if the claim is submitted within 30 days of conducting the patient interview, and if the HMR-accredited pharmacist has conducted fewer than 20 HMRs within the month. Rules state that an HMR interview must occur in the patient’s home unless prior approval has been obtained from the Pharmacy Guild of Australia, which manages the HMR program. This prior approval has to be sought by the pharmacist on a case-to-case basis, giving full patient details to the Pharmacy Guild of Australia, at least 10 days prior to the proposed interview date [4].

The evaluation of the HMR program in 2008 [5] included perspectives of GPs and pharmacists on the HMR program. Those interviewed described how while HMRs were a “good idea”, the program was not working well. Dominant themes in the evaluation report included the complexity of business rules, time delays between HMR initiation and completion, and communication difficulties between GP and pharmacist. It reported that whilst the GPs who had experienced HMRs were very positive, the others were mostly ambivalent. Many valued HMRs as a lower priority than health assessments [5].

The 2008 HMR evaluation report, commissioned by the Department of Health [5], also identified that Aboriginal and Torres Strait Islander people, despite their high burden of chronic disease, were the most likely of all Australians to miss out on HMRs and that the current HMR model was not appropriate for Aboriginal and Torres Strait Islander people [5]. A recent study [8] has explored the views of Aboriginal and Torres Strait Islander patients about the HMR program. The Aboriginal and Torres Strait Islander patients in that study felt an HMR would assist them to better understand their medicines and empower them to seek information about medicines, would improve relationships with health professionals and would increase the likelihood of medication adherence. These Aboriginal and Torres Strait Islander patients concluded, however, that current HMR rules impeded rather than facilitated HMRs for Aboriginal and Torres Strait islander people [8]. Barriers to HMR delivery were the program guidelines that stated an HMR should be delivered in the patient’s home, the referral process that required patients to organise the HMR interviews with the pharmacists and the lack of reimbursement for Aboriginal Health Worker (AHW) involvement in HMR processes [8].

This study explores the attitudes and perceptions of health professional employees working within Aboriginal Health Services (AHSs) towards the HMR program. The goal was to identify how the HMR program might better address the needs of Aboriginal and Torres Strait Islander patients. No previous HMR studies have analysed the views of health professionals working with Aboriginal and Torres Strait Islander patients.

## Method

This qualitative descriptive study explored AHS employees’ perceptions of the HMR model. The design was appropriate for this study because it facilitated the gathering of rich, contextual data related to service delivery in AHSs. Participants included GPs, nurses, AHWs and an AHS manager.

Eleven AHSs in Queensland, Northern Territory (NT), South Australia (SA), New South Wales (NSW) and Victoria participated. The sites were selected for diversity and included urban (*n* = 2), regional (*n* = 3), rural (*n* = 2) and remote (*n* = 4) settings. They varied in governance and size. Some AHSs were initiating HMRs for their patients whilst others were not. The AHSs (*n* = 5) which were known to be proactively conducting HMRs were approached so that the views of health professionals who had had experience with HMRs could be explored. The other sites were chosen to given geographical diversity. All sites approached agreed to participate.

Each AHS was given verbal, and then written information about the project, and the management and boards were asked to approve participation in the study. Each board gave written consent. Written consents were submitted to state Aboriginal ethics committees. A feedback report was sent to each AHS after research was conducted. All individual participants were given written and verbal information about the study, and written consent was obtained from each participant.

An interview guide was designed with key open-ended questions to encourage a natural exploratory conversation with the interviewee. The interviewer used the questions to prompt the sharing of the participant’s experiences and ideas. All interviews were face to face and conducted by the same researcher. Questions were modified to ensure all content raised in early interviews was explored subsequently.

Thirty-one semi-structured interviews were conducted at 11 AHSs. The numbers of each profession participating were influenced by staff availability and willingness to participate at each AHS. Fourteen AHWs, five nurses, one manager and 11 GPs were interviewed. See Table 1 for interview guide.

**Table 1:** Semi-structured Interview Guide

1. Explore attitudes to HMR program	How do you feel about the HMR program?
	How likely are you to order a HMR for a patient?
	How often do you order HMRs? What determines this?
2. Explore understanding of HMR processes	Who do you order HMRs for? Why?
	How do you find the HMR process?
	Do you have assistance from other staff members in organizing HMRs? If so, how?
3. Identify reasons for ordering HMRs (benefits)	How useful have you found HMRs? or How useful do you think an HMR could be?
	What is the most useful aspect of an HMR?
	What feedback have you had from your patients about the HMR?
	How do you find the pharmacists’ reports?
4. Identify barriers to initiating HMRs	Is there a reason why you don’t order more HMRs? Please explain
	Would you like to order more HMRs? Please explain
	What are the limiting factors in referring patients for an HMR?
	Why do you think there are not many HMRs are being conducted for Aboriginal and Torres Strait Islander patients?
5. Encourage recommendations	Do you believe the current HMR model is effective/not effective? Please explain
	How appropriate is the HMR model for your patients?
	Are there any ways the model could be improved? If so, how?

Of the 11 participating AHSs in this study, three were conducting HMRs regularly, four occasionally and four not at all. Only at the three AHSs, where there were contracted pharmacists, were patients being referred regularly for HMRs. One of these AHSs had a salaried pharmacist employed by the AHS for a range of clinical pharmacy roles, including HMRs. The other two had each contracted an HMR-accredited pharmacist to conduct HMRs, with one using a chronic care nurse and the other an AHW to co-ordinate the program. Although only three GPs were referring patients for regular HMRs, all interviewed GPs were aware of the HMR program although some lacked understanding of the HMR referral processes. The majority of nurses and AHWs interviewed were unaware of the HMR program.

Interviews were recorded, de-identified and transcribed verbatim. Transcripts were coded and analysed for themes that recurred throughout the interviews. Analysis occurred concurrently.

Ethics approval was sought and granted from the University of Sydney Human Research Ethics Committee (11504), the Aboriginal Health and Medical Research Council (NSW), the Menzies School of Health Research (NT, SA) and the Aboriginal Health Research & Ethics Committee (Victoria).

## Results

The study participants who had experienced an HMR were extremely supportive of the program. The four GPs who had never referred patients for HMRs expressed reservations about the value of HMRs and concerns over the need to burden patients with further referrals. Two of the nurses interviewed were not supportive of HMRS. These nurses believed that although HMRs were “good in theory” Aboriginal patients were “not interested” and “there’s no point filling them up with a huge amount of education if they are not going to take the medicines anyway”. The AHSs who had not previously been involved in HMRS were very keen to understand the details of the HMR program as they felt it would greatly assist their clients who they believed often “don’t understand how the medicines work, when they work, and they don’t take them at the right times or the right way”. Most of the interviewees expressed positive views regarding the potential benefits of HMRs for their patients’ health.

The emergent themes, and the perceived benefits of and barriers to the HMR program, are discussed below and summarised in Table 2.

**Table 2:** Most common perceived benefits and barriers of the HMR program

Benefits of HMRs	AHS staff comments
Increased patient understanding and confidence	*The HMR interview is a good opportunity to iron out some confusion about medicines. (AHW)*
	*The clients need to know the importance of taking medicines and why they are taking them. (AHW)*
	*It helps my patients understand their medicines a bit more. (GP)*
	*Just having another person go over it, having a bit more time and in different words can be very useful. (GP)*
	*There’s the empowerment they(the patient) get from a more clear understanding. (nurse)*
Improve medication adherence	*Because the people don’t feel they’re working, they tend not to take them. (AHW)*
	*If you explain to them (the patients) what it is, how it works and what to watch out for, then there’s some informed decision making and they’re more likely to take them (medicines). (AHW)*
	*It gave my patient more confidence to take his medicines, just having someone reassure him that the medicines he was taking were appropriate. (GP)*
Supporting GP practice	*You get to learn stuff that you wouldn’t normally know about your patient. You learn about the gap, about what you think is going on and what is really going on, and you also learn stuff about medicines that you didn’t know*. (GP)
	*The reports can be revelationary. You find out people are taking all sorts of things, some that you ceased months ago. (GP)*
	*When a locum comes, and we have lots, they just prescribe the drugs because the patient asks for them. They don’t review them or work out if they really need them. (AHW)*
Barriers to HMRs	AHS staff comments
Lack of awareness	*None of us here know about home medicines review. (AHW)*
	*People are not aware they can ask for, or should ask for their medicines to be reviewed. (AHW)*
	*They (the patient) don’t know that pharmacists can do things like reviews. (AHW)*
Workload	*Time is the main thing that has put me off (GP)*
	*We are already inundated with administrative tasks (GP)*
	*Aboriginal Medical Service workloads are pretty demanding. A lot of these people that qualify for an HMR also qualify for EPC, care plans, health assessments and that kind of stuff, so that might be where they’re going first. (nurse)*
	*One of the difficulties is having enough health workers on board to do it (participate in an HMR). Having a health worker who is trained enough to go with the pharmacist, who is trained in quality use of medicines and who understands what the pharmacist is talking about and take a lead in the whole process would be the ideal. (AHS manager)*
Protection of the Clinician-client relationship	*They’re (patients) already getting referred to lots of different people for lots of different things. So another referral might just feel like too much (GP)*
	*Gaining someone’s confidence and trust and having a meaningful clinical interaction requires proper cross cultural training and working with the community over some time.(GP)*
	*Doctors are concerned about overloading the patient. (nurse)*
Lack of Clinician/AHS pharmacist relationship	*The GPs aren’t driving it (HMR referrals) as they don’t have a relationship with a pharmacist who can do it for them (GP)*
	*The relationship between the doctor and the pharmacist might not be established. If they had a rapport and a referral pathway going already that would really help. (nurse)*
	*The community pharmacists around here are very busy. I don’t think they have time to get it done (GP)*
	*It would be important for the pharmacist to have some cross cultural training (AHW)*
	*The chronic health nurse or AHW needs to have a direct link with the accredited pharmacist, not the pharmacy. (nurse)*
	*Generally our clients do not have a relationship with a pharmacist (nurse)*
Lack of an HMR facilitator/driver/ program manager	*We need someone at the health service allocated to encouraging the home medicines review, co-ordinating it, blocking out time for GPs to do referrals, taking on the role of doing the consent. (GP)*
	*It needs something set in place so that it can be done regularly (GP)*
	*We rely on a co-ordinator to organize all the logistics (GP)*
	*There needs to be a single point of contact, health worker to patient.(AHW)*
	*Somebody who is well known to the patient needs to ring and explain the process. (AHW)*
Complex HMR model and rules	*It took a while to make sense of the steps (GP)*
	*I think the criteria are a bit restrictive. (GP)*
	*It was not clear that all pharmacists were not accredited. I was sending off referral letters and nothing happened (GP)*
	*It would be better if someone else could refer. For a multidisciplinary team to work effectively everything should not be done by the GP. (GP)*
	*A lot of them think it is all done once the pharmacist has left the house. (AHW)*
	*The health service should promote it (HMR) and align it with other programs or something they do already.(nurse)*
	*We don’t organize home medicine reviews for all sorts of reasons – around privacy, judgment, people not being home, lots of people being transient or homeless, lots of people in 1 household and people not wanting strangers in their home. (nurse)*
	*We need a flexible mixed model where some people can come here on an appointment, or we can go there if it suits today or where a pharmacist can just add on to an existing program. (AHW)*
Lack of Financial Reimbursement	*The AMS should be able to claim something for organizing an HMR. (AHS manager)*
	*It should be the AMS who is doing all the organizing who gets a cut, not the pharmacy. (nurse)*
	*AHWs are very important to the process. They need to be reimbursed for their time, just like the pharmacists and GPs. (GP)*

### “Home Medicines Reviews are useful”

Three of the five nurses and all AHW interviewees described increased medicine knowledge and empowerment of patients to make medicine choices as the potential benefits of the program*,* stating, “HMRs were good for understanding what they’re taking and why they are taking medicines and the importance of medicines”. They also felt HMRs would assist patients to learn about potential dangers in storing medicines and sharing medicines. Most of the interviewees strongly agreed that an HMR could be useful in reducing medication “fear and worries about the unknown”.

All the AHW interviewees expressed concern about patient confusion regarding their medicines, stating “generics confuse the hell out of people” and “in hospital they start swapping and changing medications. It gets very confusing”.

The majority of the GP interviewees also felt patients would benefit from increased medicine knowledge and that patients would benefit from having “someone else reinforcing information that the doctor has given”. The majority of GPs believed that HMRs could assist their patients to feel more confident about taking their medicines and felt HMRs would “elevate the medications up the priority list”. The majority of participants believed that most patients would be “really keen” to have HMRs, although there may be a few patients who “see it as a failure to have someone come and talk to them”.

The GP interviewees who had referred patients for HMRs praised how HMRs had identified potential drug interactions and had identified “an astounding number of discrepancies between what we had on our system and what clients were taking”*.* Also, these GPs valued how HMRs assisted their therapeutic decision-making, assisted them to sometimes cease medications and increased their own understanding of medicines. The GPs liked the HMR reports as the “pharmacists fed back lots of information about whether there are lots of other medications from other places and whether there is confusion and that sort of thing”.

Other benefits of HMRS stated by the GP interviewees included improved understanding of whether their patients had high falls risk, were medication adherent, and were sharing or hoarding medicines. A few GP participants also commented that they felt HMRs would assist with building relationships between patients and pharmacists.

### “I just don’t get around to ordering HMRs”

Despite most of the participating AHS GPs agreeing that HMRs would be very useful for their complex patients and for supporting their therapeutic decisions, only three of the 11 GPs interviewed were actually referring their patients for HMRs regularly.

The most common reasons for the GPs not “getting around” to ordering HMRs for their patients included lack of time, protection of their client-clinician relationships, lack of relationships with pharmacists and cultural inappropriateness of the HMR program. Some other reasons included complex HMR processes, not prioritising medicines in their patient discussions, GP ownership of their role in advising on medicines, and perceived lack of evidence for the value of HMRs. Two of the GPs reflected paternalistic attitudes, commenting, “they [their Aboriginal patients] are not particularly interested in having an intervention like a HMR” and “In terms of education, which I know is one of the really important parts, I’m not sure. I’m not convinced that those people think it is a high priority and that we have any way of educating these people about their medications”. Two GPs commented that reviewing patients’ medicines was part of their practice, stating, “if a patient had concerns about their medicines they would come and talk to us about it. The clinic has primary responsibility for those things” and “I do a lot of it [medication review] myself actually”.

All the GP interviewees commented that the biggest barrier to writing HMR referrals was “being pushed for time”, and half of the GPs felt that writing an HMR referral was a barrier as it was “just another bureaucratic, red tape thing to do when you’re seeing patients”. Patients at the AHSs often had complex co-morbidities, and although most interviewees agreed that HMRs were desirable, the GPs felt they had “to sort out the multiple things a patient presents with, do a GP plan and a team arrangement and a health check first. An HMR is just another thing on a list of things that you know you need to do”. At times some felt they were “snowed under with the acute stuff before you even get to the chronic stuff”. In the three AHSs where GPs were writing regular HMR referrals they had found other staff members to assist the process, and one commented, “It takes time to offer and explain it [HMR] and do the referral. That is just too onerous to fit into an appointment. So I get the health worker to do it.”

The eight GPs who were not regularly referring patients for HMRs made comments which reflected their wish to protect their clinician-client relationships. “There are lots of practitioners with lots of clinicians involved already” and “We need to make sure we are not overburdening them [the patients] with our efforts.” These GPs particularly showed some uncertainty about referring their patients to a pharmacist, indicating a lack of GP-pharmacist relationships. Five GPs perceived that their local pharmacists were too busy to do HMRs, commenting, “we think that some pharmacists are too busy. I guess we worry that the pharmacist might not be very receptive.” Two GPs had had their HMR referrals ignored or returned by pharmacies. “I was sending off referral letters and nothing happened.”

Seven of the GPs commented that pharmacists needed to be culturally sensitive, have some cultural training and/or show an interest in working with Aboriginal people before they would feel comfortable referring their Aboriginal patients to them. Comments included, “I am not sure how culturally aware the pharmacists are” and “If we [the AHS] had a relationship with a particular pharmacist who we knew our people were comfortable with that would really help.” The nurses also felt that the lack of a pharmacist-client relationship was problematic, stating, “It’s not very often that you will have a relationship between the client and the pharmacist.” Conversely, in the AHSs where pharmacists had been contracted, the pharmacists were highly valued and regarded. “They [the GPs] really like having the pharmacist here. The doctors specially allocate time when she is here for the day. Now that they have built up a rapport the doctors will actually ring her up and ask her questions about medications.” The AHWs also commented, “It would be important for the pharmacist to have some cross-cultural training. For them to be good at it, for it to be worthwhile, they need proper cultural training. That would be key.” A few of the interviewees bemoaned the lack of Aboriginal or Torres strait Islander pharmacists. The majority of AHWs commented on their clients’ lack of understanding of the pharmacist’s role and on their lack of established relationships with the local community pharmacists.

The GPs, nurses and AHWs all showed some misgivings about a pharmacist visiting an Aboriginal or Torres Strait Islander person’s home. “I think sending a pharmacist cold to a patient’s house is inappropriate,” and “many Aboriginal people are not comfortable with non-Aboriginal people going to their home.” Half the GPs did, however, state a preference for the HMR interview being conducted at the patient’s home whenever possible as they cited the advantage of being “able to see what is really happening” and that “one of the great benefits is seeing the context at home. So it would be a shame to lose that”. However, most felt that although “It is better if it happens in the home. I wouldn’t want them to not get an HMR just because the home is not appropriate or suitable”, and some believed that it was preferable to “have something in the clinic where they’re used to coming”. A couple of interviewees also commented that having a pharmacist at the clinic would assist in establishing relationships among GPs, pharmacists and AHWs and would allow valuable case conferencing and discussion about patients.

Despite most of the GPs at first stating that HMRs could be beneficial in assisting their patients to manage their medicines, later in their interviews, half the GPS from the AHSs where HMRs were not occurring, showed some scepticism about the value of HMRs. Two of the 11 GPs perceived that “if patients had concerns about their medicines they would come and talk to us (GPs) about it”. These GPs appeared to doubt the need for HMRs as they felt they adequately dealt with medication issues themselves, saying, “I do a lot of it [medication education] myself actually” and “I believe it is my role to talk to patients about their medicines.” Three GPs also expressed concern that their patients “might not see the value in it” and stated, “the doctors spend a lot of time dealing with medicines. So it might be seen as doubling up.” The majority of GPs who were not ordering HMRs felt that currently “the process isn’t in place for it to happen”.

The AHWs perceived that their lack of awareness and their clients’ lack of awareness of the HMR program contributed to the low uptake of HMRs by their communities. Some also commented on the lack of continuity of GPs and the number of health checks already being conducted as barriers to implementing another program, such as HMR, in their health services.

### “Need someone to be the main organiser”

All the AHS health professionals interviewed agreed that for HMRs to become a regular occurrence at their AHSs it required “having someone at the health service allocated to encouraging the HMR, co-ordinating it, blocking out time for GPs to do the referral, taking on the role of doing the consent”. They all agreed that this role should be done by a senior health worker or a nurse who really understands the process. Each of the three AHSs where HMRs were being done had a “co-ordinator to organise all the logistics”. One AHS used the chronic care co-ordinator nurse, another an AHW dedicated to Quality Use of Medicines and the third a salaried pharmacist to organise their HMRs.

Explaining the process to the patient and brokering trust in the process was seen as an important in the success of the program with Aboriginal and Torres Strait Islander patients. The GPs explained, “Someone who is well known to the patient, such as a health worker, needs to ring and explain the process,” and “I think if the health worker is the first port of call and clearly explains everything, then I think people will take it up.”

Across the health professional groups there was discussion regarding how best to manage the extra work load that HMRs would create. Most agreed that there should be health workers specifically employed as chronic disease health workers or even specifically as medication specialists, and part of their role should be facilitating the HMR process. All agreed,”the AHS should be able to claim something for their [the organiser’s] time” and that “it should be the AHS who is doing all the organising who gets a cut, not the pharmacy”.

Most GPs agreed an AHW should accompany a pharmacist to a client interview to broker cultural trust because “it provides an opportunity to up-skill the health worker”. Most commented that the health worker or the AHS should receive financial reimbursement for the health worker’s time, in alignment with the fees received from the Government by the GP and the pharmacist. Many interviewees felt the organising health workers should also be the ones attending the interviews.

### “It would be better if someone else could refer”

A few of the GPs showed a lack of confidence and knowledge about who was eligible for an HMR, about how to write a referral, and about the HMR process itself, as indicated by their comments, “so you don’t have to wait until they are on 5th medication to order an HMR” and “It takes a while to make sense of the steps”. All the GPs, nurses and AHWs agreed that “it is not practical for the patient to have to take the referral to the pharmacy”. Most of the GPs indicated that as they were time-poor, they would be happy for a health worker or nurse to organise the HMR and even write the referral, or alternatively “we could do the referral retrospectively”. The AHWs iterated their willingness to initiate referrals: “we know the patients best. So it would make more sense if we organised the referral.”

The nurses and AHWs believed that it was crucial that the AHS select and refer to a specific pharmacist known to the AHS, with whom they had a relationship and who had been assessed by the health service for their cultural sensitivity. The GPs also expressed the need to establish rapport with a trusted and culturally appropriate pharmacist before they would refer their patients.

### “The model needs to be changed”

In addition to changing referral pathways, having the AHS organise the HMR, and having an AHW attend the HMR interview, GPs also suggested other changes to the HMR model. These included the pharmacists providing patients with a brief follow-up report that also prompted the patient to make an appointment with the GP to discuss the report, as “it is a very important step when the patient sits down with the GP and makes the changes that are needed”. Many lamented that often HMR patients did not revisit the clinic to discuss a revised medication plan with the GPs.

Half the study participants mentioned that HMRs should be incorporated into the Aboriginal health assessment process, or be part of the existing GP management plan. A number of health workers also stated that the HMR program would work best if it was a “flexible model where you can work in with existing programmes rather than trying to develop a whole other system of doing things”. The AHWs suggested pharmacists should join in existing groups, run by the AHS, such as cardiac rehabilitation, cooking or diabetes groups or “run alongside a chronic disease clinic that’s happening on the day”. Most of the AHWs mentioned that group meetings would be favoured by many clients, and so group HMRs should be an option.

All interviewers agreed that for the HMR program to work within their AHSs it needed to be simplified. At present there are “way too many steps”. It also needed a systematised approach to ensure HMR referrals were written, interviews organised, and patients followed up. “It needs something set in place so that it can be done regularly.” The HMR system required a “driver” who was not too overburdened with other duties, preferably an AHW dedicated to chronic disease and medicines. All the AHWs interviewed stated that advising patients about medicines was a key part of their role and that they would like more training in this area.

The AHWs suggested that AHSs need to promote the HMR program and inform their clients of the steps involved, through pamphlets and posters in the AHSs.

The GPs and AHWs suggested changing the name of the HMR program. “The title is not good as some patients don’t like that home bit. Some don’t like strangers coming to their home. It needs an Aboriginal title or at least a bit more of a friendly title.”

**Fig. 1:**
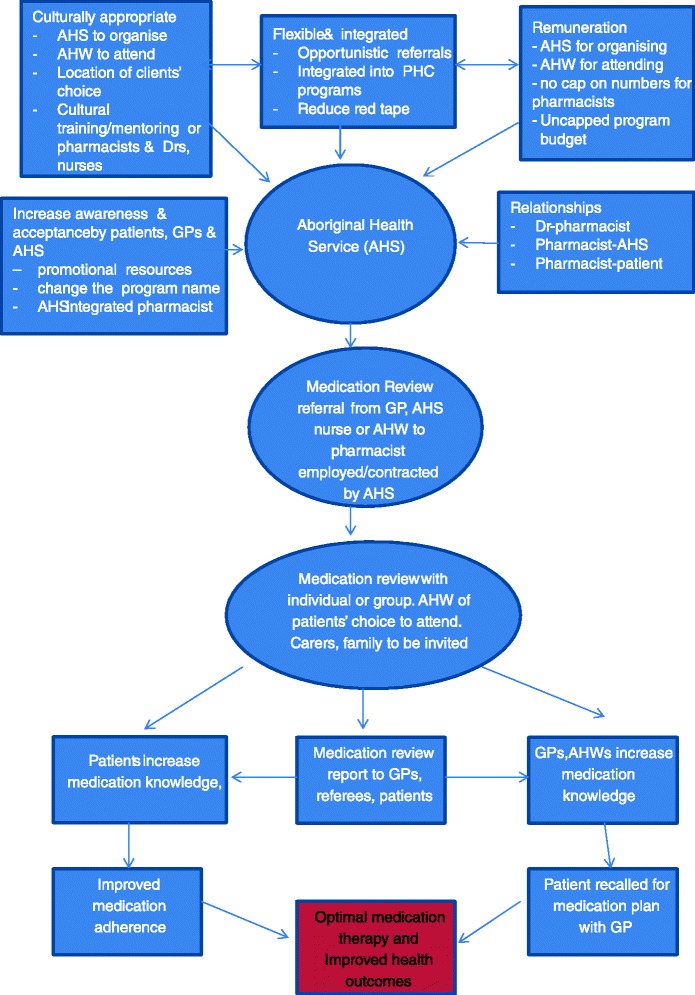
Suggested model for medication review for Aboriginal and Torres Strait Islander people

Figure 1 summarises the recommendations for a revised, more culturally appropriate HMR model. It is hoped that the findings and recommendations from this study will inform the Sixth Community Pharmacy agreement on HMR program rules for Aboriginal and Torres Strait Islander people.

## Discussion

Previous research has shown that consumers identified improved medicine information, feeling cared for, and increased confidence to discuss medicines with their GPs, as potential benefits of an HMR [8, 9]. In this study, the perspectives of the nurses and GPs who had experienced HMRs, and the perspectives of all the AHWs, strongly supported the findings from research with consumers, as they, too, identified that increased medicine knowledge and empowering consumers to make medicine choices were the major HMR benefits. Although the majority of GP participants, especially those who had experienced HMRs, agreed that HMRs could assist their understanding of their patients’ medicine practices and provide clinical support, very few HMRs were being ordered. The GPs were only referring their patients for HMRs in three out of the 11 AHSs in this study.

The 2008 Campbell report identified that GPs need assistance with the structure related to HMRs and that an HMR is very dependent on the relationship between GP and pharmacist [5]. This study reinforced this need for structure and relationship, with HMRs only occurring in AHSs where structure had already been established, with an AHS staff member “driving” the process and where a pharmacist-AHS relationship had been established. This study also reinforced the Campbell report findings that an HMR was not seen as being high on the list of priorities for GPs, due to competing demand for GP time; as a result the HMR program existed in isolation rather than in parallel with other Medicare items [6]. The participants in this study reiterated that the HMR program workload needed to be shared and a team approach adopted, especially in areas where there were medical workforce shortages.

This study confirmed a number of the barriers to provision of HMR services identified in previous studies by both consumers and stakeholders. These included complexity of program rules, concerns regarding home visits, lack of information about the program, GP workload and GP fears of pharmacists encroaching on their professional space [5, 7–9]. It also identified a number of barriers specific to Aboriginal and Torres Strait Islander clients. These included paternalistic attitudes of health professionals to clients, heightened protection of the GP-client relationship, lack of AHS-pharmacist relationships, need for more culturally responsive pharmacists and the lack of recognition of the AHS’s role in implementation of culturally effective HMRs.

One quarter (*n* = 4) of the non-Indigenous health professionals (*n* = 16) interviewed, demonstrated paternalistic or racist attitudes to their clients, claiming that their clients were uninterested or incapable of learning more about their medicines and thus not suitable for HMRs. This directly contradicts research conducted with the Aboriginal and Torres Strait Islander patients and the AHWs at the same AHSs. The majority of patients at these AHSs were extremely interested in learning more about their medicines and very supportive of having HMRs [8, 10]. The AHWs strongly believed their clients would benefit from HMRS as long as the HMRs were conducted in a culturally appropriate manner. The small sample of non-Indigenous health professionals interviewed does not allow for extrapolation across the AHS workforce but does support previous work which suggests that GPs and non-Aboriginal staff at AHSs would benefit from cultural mentoring [11]. These attitudes require further investigation to assess whether some health professionals at AHSs may require screening or further cultural training.

The majority of the GPs interviewed in this study were very protective of their client-clinician relationships, with much of the GP concern related to not overloading the patient with information and too many appointments. Further research is needed to ascertain whether this concern about “overloading” the patient is culturally influenced. There was also considerable concern from the AHS GP and nurse interviewees that pharmacists may be culturally insensitive and thus, by association, may damage patient trust. Only at the AHSs (*n* = 3) where a pharmacist was contracted or embedded was there a real understanding of the clinical role of the pharmacist. The lack of relationships of the AHS staff, including the GPs, with any pharmacists, including their local community pharmacists, appeared to be a major barrier to the initiation of HMRs. The lack of relationships with AHSs was also noted by pharmacists themselves in recent research [7]. This supported previous research which suggested that lack of face-to-face interactions and established relationships between GPs and community pharmacists may be significant barriers to collaboration [12]. It appears that significant work is needed to build bridges between pharmacists and GPs, and between pharmacists and AHSs, and to provide cultural training for pharmacists. Pharmacy organisations are currently lobbying the Commonwealth Government to fund salaried pharmacists within AHSs to enable culturally trained pharmacists to develop relationships with AHSs and their clients [13].

All the interviewees agreed that for many of their Aboriginal and Torres Strait Islander clients to feel confident in engaging with HMRs, the HMRs needed to be organised and facilitated by AHS employees. This was also the finding of recent research which examined the views of Aboriginal and Torres Strait islander patients who also identified the need for the option of having an AHW attend the HMR interview [8]. Despite the acknowledgment by the Australian Government that pharmacies organising HMRs are entitled to a fee [4], there has been no acknowledgment of reimbursement for AHSs, which fulfil an even larger role in HMR facilitation for Aboriginal and Torres Strait Islander people.

The AHWs in this study identified the need for AHSs to have AHWs who specialise in assisting patients with medication management and who could facilitate the HMR process. Most AHWs were keen to undertake further training about medications as they saw assisting patients with their medications as an important part of their role. In the AHSs (*n* = 3) which were initiating HMRs, a number of the AHWs were identifying patients and organising HMR referrals. All interviewees, including the GPs, were keen for nurses and AHWs to be allowed to write HMR referrals, seeing it as unnecessary for GPs to be involved in this process. Another study has requested that community nurses be allowed to refer patients for HMR [14]. A one-off HMR every 12–24 months was not seen as ideal. For complex patients with multiple medications, regular interactions with pharmacists to reinforce medication messages is needed.

Despite the Australian Government’s commitment to improving the health and wellbeing of Aboriginal and Torres Strait Islander Australians and to closing the health inequality gap [15], Aboriginal and Torres Strait Islander health status remains poor, and burdens of chronic diseases, such as respiratory disease, diabetes and cardiovascular disease, remain very high [16]. A number of studies have identified that medication management is an important issue which urgently needs to be addressed if the progression of chronic disease and all the associated complications are to be slowed [10, 17–19]. Although the Australian Government has implemented a number of programs to assist Aboriginal patients with financial barriers to accessing medicines, recent changes to the HMR program rules have increased the barriers to accessing HMRs, and thus exacerbated issues of medication management, efficacy, safety and adherence. Changes to the HMR program have purposively been implemented to curtail the number of HMRS being conducted to reduce expenditure in a program with a capped budget [4]. These program changes have disproportionately affected those most in need, i.e. Australia’s sickest people, the elderly, rural people and Aboriginal people [6].

The VALMER study was an economic evaluation of the HMR program by the University of Tasmania, which analysed 180 HMRS across Australia. It concluded that HMRs could significantly decrease healthcare utilisation costs as well as improve patients’ quality of life [20]. Overall healthcare savings and benefits should be taken into consideration when funding for the HMR program is assessed and guidelines rewritten in the Sixth Pharmacy Community Agreement 2015. New health models, such as shared medical appointments, which use group consultations to improve patient health, should be used to inform new HMR modelling and maximise outcomes from expenditure [21, 22]. Recommendations from this study and from the 2008 Campbell evaluation report should be considered in developing an HMR model which is effective and culturally appropriate for Aboriginal and Torres Strait Islander people.

### Study limitations

The sample of AHS health professionals who were interviewed was small, and the representation of each profession even smaller, and therefore findings cannot be extrapolated to all AHS employees or across professions. The views of health professionals who work with Aboriginal and Torres Strait islander people in settings other than AHSs were not sought. However, internal validity and reliability was achieved by asking questions about the same issues numerous times, in appropriate, non-leading ways, with this method yielding similar findings in a range of different settings. Many of the findings in this study endorsed results from research undertaken with Aboriginal and Torres Strait Islander patients [8] and pharmacists working with AHSs [7].

## Conclusion

Increasing HMRs for Aboriginal and Torres Strait Islander people has the potential to increase patients’ medication knowledge and medication adherence and thus improve chronic disease management [8]. The HMRs teach health service staff about their patients and about medications, and provide GPs with invaluable information which assists them to more optimally manage their patients’ medications and health.

Currently, very few HMRs are being conducted with Aboriginal and Torres Strait Islander people, largely due to lack of awareness, the paternalistic attitudes of some health professionals and the logistics of navigating the HMR program rules. The GPs at most AHSs are writing very few HMR referrals due to the complexities of patients’ needs, shortage of time, and lack of trust in pharmacists’ ability to appropriately manage their patients.

The AHSs, as the trusted brokers of Aboriginal social, emotional and physical wellbeing and with their understanding of community history and relationships, are well-placed to be the promoters, organisers, facilitators and implementers of health programs, such as HMRs, for Aboriginal and Torres Strait Islander clients. Within AHSs, staff juggle numerous programs and funding streams, and so the HMR program needs to be simplified and integrated within existing programs, and have “champions” in each health service to promote and drive the program.

The name of the HMR program and the myriad of HMR rules need to be changed and simplified. Referral, feedback and follow-up processes in particular need to be revised. Much work is needed to improve GP-pharmacist professional relationships and pharmacist-AHS relationships. The GPs, nurses and AHWs who have no previous experience with HMRs have little or no understanding of the role of the pharmacist. A big-picture approach is needed in the restructuring of the HMR program, using evidence-based decision-making and Aboriginal community consultation.

Embedding pharmacists within AHSs, even in a part-time capacity, is a solution which addresses many of the barriers to HMRs which have been identified in this study. It enables the HMR program to be integrated with other services and assists GPs to offer optimal medication therapy. It ensures pharmacists are culturally mentored and that they build strong relationships with health professionals and patients. It encourages regular “coaching” of patients to assist medication adherence. If the Australian Government is to honour its commitment to improve Aboriginal and Torres Strait Islander health it needs to fund an uncapped medication review program and embed salaried pharmacists within AHSs.

## Abbreviations

AHS: Aboriginal Health Service
AHW: Aboriginal Health Worker
GP: General practitioner
HMR: Home Medicines Review
MR: Medication review
